# Anti-cyclooxygenase, anti-glycation, and anti-skin aging effect of *Dendrobium officinale* flowers’ aqueous extract and its phytochemical validation in aging

**DOI:** 10.3389/fimmu.2023.1095848

**Published:** 2023-03-17

**Authors:** Huiji Zhou, Luxian Zhou, Bo Li, Rongcai Yue

**Affiliations:** ^1^ Amway (Shanghai) Innovation and Science Co., Ltd, Shanghai, China; ^2^ Shanghai Archgene Biotechnology Co., Ltd, Shanghai, China; ^3^ Amway (China) Botanical R&D Center, Wuxi, China; ^4^ School of Pharmacy, Fujian Medical University, Fuzhou, Fujian, China; ^5^ Fujian Key Laboratory of Drug Target Discovery and Structural and Functional Research, Fujian Medical University, Fuzhou, Fujian, China

**Keywords:** *Dendrobium officinale*, flavone di-C-glycosides, UPLC-ESI-qTOF-MS/MS, anti-aging, antioxidant

## Abstract

**Introduction:**

*Dendrobium officinale* Kimura et Migo (*D. officinale*) , widely called as “life-saving immortal grass” by Chinese folk, is a scarce and endangered species. The edible stems of *D. officinale* have been extensively studied for active chemical components and various bioactivities. However, few studies have reported the well-being beneficial effects of *D. officinale* flowers (DOF). Therefore, the present study aimed to investigate the in vitro biological potency of its aqueous extract and screen its active components.

**Methods:**

Antioxidant tests, including 2,2-diphenyl-1-picrylhydrazyl (DPPH), 2,2′-azino-bis(3-ethylbenzothiazoline-6-sulfonic acid) (ABTS), the ferric reducing ability of plasma (FRAP), and intracellular reactive oxygen species (ROS) level analyses in primary human epidermal keratinocytes, anti-cyclooxygenase2 (COX-2) assay, anti-glycation assay (both fluorescent AGEs formation in a BSA fructose/glucose system and glycation cell assay), and anti-aging assay (quantification of collagen types I and III, and SA-β-gal staining assay) were conducted to determine the potential biological effects of DOF extracts and its major compounds. Ultra-performance liquid chromatography-electrospray ionisation-quadrupole-time-of-flight-mass spectrometry (UPLC-ESI-QTOF-MS/MS) was performed to investigate the composition of DOF extracts. Online antioxidant post-column bioassay tests were applied to rapidly screen major antioxidants in DOF extracts.

**Results and discussion:**

The aqueous extract of *D. officinale* flowers was found to have potential antioxidant capacity, anti-cyclooxygenase2 (COX-2) effect, anti-glycation potency, and anti-aging effects. A total of 34 compounds were identified using UPLC-ESI-QTOF-MS/MS. Online ABTS radical analysis demonstrated that 1-O-caffeoyl-β-D-glucoside, vicenin-2, luteolin-6-C-β-D-xyloside-8-C-β--D-glucoside, quercetin-3-O-sophoroside, rutin, isoquercitrin, and quercetin 3-O-(6″-O-malonyl)-β-D-glucoside are the major potential antioxidants. In addition, all selected 16 compounds exerted significant ABTS radical scavenging ability and effective AGE suppressive activities. However, only certain compounds, such as rutin and isoquercitrin, displayed selective and significant antioxidant abilities, as shown by DPPH and FRAP, as well as potent COX-2 inhibitory capacity, whereas the remaining compounds displayed relatively weak or no effects. This indicates that specific components contributed to different functionalities. Our findings justified that DOF and its active compound targeted related enzymes and highlighted their potential application in anti-aging.

## 1 Introduction

Skin is mainly composed of several structures, including the epidermis and dermis, and protects the body against sunlight radiation. The epidermis, the outmost layer of the skin, mainly consists of keratinocytes and absorbs most of the ultraviolet B (UVB) irradiation (1). UVB irradiation stimulates the overproduction of various reactive oxygen species (ROS), which can trigger complex inflammatory signal cascades, such as aberrant cyclooxygenase-2 (COX-2) expression (2), and ultimately lead to various adverse symptoms, including wrinkle formation, skin sagging, and dryness. The dermis, a support tissue maintaining skin resistance and elasticity, harbours critical skin cells referred to as fibroblasts, which are responsible for the synthesis and secretion of the extracellular matrix (ECM) (3). Collagen, mainly types I and III, is the most abundant fibrous protein found within the ECM. However, collagen degradation occurs with age, and its content decreases at a rate of approximately 1% annually after the age of 20, leading to wrinkles, sagging skin, and premature aging (4). Additionally, increased cellular senescence has been found to promote the aging process. Senescent cells exhibit various distinctive molecular properties; senescence-associated *β*-galactosidase (SA-*β*-gal) is one of the best-characterised and frequently used biomarkers to identify senescent cells (5, 6). Normal cells have a limited capacity to replicate and eventually enter the senescent state, in which SA-*β*-gal activity increases rapidly (7, 8).

Furthermore, superfluous reactive free radicals produced during the oxidation process induce abnormal protein modification, destroy the secondary structure, and ultimately accelerate the formation of advanced glycation end products (AGEs), which stimulate ROS production. AGEs are a series of compounds gradually formed as a result of a non-enzymatic glycation reaction with fluorescent and non-fluorescent entities, such as argpyrimidine and *N*-carboxymethyl lysine (CML) (9). Accumulation of AGEs occurs throughout life, and they are found at significantly higher levels among the elderly population (10). Growing evidence has shown that AGEs are a major etiologic factor in age-related disorders, especially skin aging, as it is the most direct manifestation of body aging. A few studies also have shown that external stimuli such as excessive free radicals and spontaneous AGE generation *in vivo*, which are irreversible once formed in the body (11), are associated with skin fibroblast damage, destruction of collagen and elastic fibres, a yellow complexion without splendour, and deterioration with aging (12). Therefore, antioxidant, anti-inflammatory, and anti-glycation effects, as well as collagen protection and SA-*β*-gal inhibition, are important for the development of anti-aging products.

*D. officinale* Kimura et Migo, widely referred to as “life-saving immortal grass” by the Chinese, is a scarce and endangered species due to its unique environmental requirements, low fertility, slow growth, and sparse distribution (13). The edible stems of *D. officinale*, known as “TiepiShihu”, have been extensively studied in recent years for active chemical components (14) and used as a precious medicinal herb to improve immune function, nourish the stomach, alleviate the symptoms of diabetes, and postpone senility (15). However, as a medicinal by-product, the flowers of *D. officinale* are typically discarded and rarely studied. Recently, the flowers of *D. officinale* have been reported to be rich in nutrients and other chemical ingredients, including anthocyanins, flavonoids, and polysaccharides, and specific bioactivities have been elucidated, such as liver protection as well as hypoglycaemic, antioxidant, and antihypertensive effects (16–18). Therefore, it is necessary to establish reliable methods for component analysis of *D. officinale* flowers and expand its use for health-related products through comparison with stems.

The *D. officinale* flower is popular in China due to its anti-aging properties and beneficial effects on yin deficiency syndromes. However, scientific data to confirm the pharmacological effects listed above is limited in the literature. Therefore, the purpose of this study was to tentatively conduct more systematic experiments on the aqueous extracts of *D. officinale*, including its biological effects on skin aging, such as antioxidant capacity (through 2,2-diphenyl-1-picrylhydrazyl (DPPH), 2,2′-azino-bis(3-ethylbenzothiazoline-6-sulfonic acid)(ABTS), the ferric reducing ability of plasma (FRAP)), anti-inflammatory effect (through COX-2), anti-glycation potency (through inhibition of non-enzymatic glycation reaction and inhibition of CML expression in fibroblasts), and anti-aging evaluation (through the SA-*β*-gal staining test and collagen expression). The phytochemical composition of *D. officinale* was also analysed using ultra-performance liquid chromatography-electrospray ionisation-quadrupole-time-of-flight-mass spectrometry (UPLC-ESI-QTOF-MS/MS). Antioxidants in *D. officinale* flower aqueous extract were identified using the online ultra-performance liquid chromatography-photodiode array detection-mass spectrometry-2,2′-azino-bis(3-ethylbenzothiazoline-6-sulfonic acid (UPLC-PDA-MS-ABTS+·) method and the biological capacities of specific isolated constituents were explored (through DPPH, ABTS, FRAP, anti-COX-2, and AGE inhibitory activities). Previous research has shown that *D. officinale* flowers can alleviate brain aging and improve spatial learning abilities in senescent rats (17). However, previous literature contains few reports on the anti-glycation effect of *D. officinale* flower aqueous extract. Online antioxidant investigation of *D. officinale* flower aqueous extract and various biological activities of its identified compounds that are described in the work have not been reported previously.

## 2 Materials and methods

### 2.1 Plant material, solvents, and chemicals

The flowers of *D. officinale* were purchased by Amway (Shanghai) Technology Co. and authenticated as *D. officinale* Kimura et Migo flowers by doctor Gangqiang Dong, Amway (China) Botanical Research Centre. Analytical-grade methanol was purchased from Honeywell Co. (Charlotte, USA). Acetic acid and liquid LS-MS-HPLC-grade acetonitrile was purchased from Merck (Darmstadt, Germany). Bovine serum albumin (BSA) lyophilised powder, ABTS (98%), DPPH· (98%), 6-hydroxyl-2,5,7,8-tetra-methylchroman-2-carboxylic acid (Trolox), tyrosinase, ascorbic acid, NS-398 (COX-2 inhibitor, CAS: 123653-11-2), and dimethyl sulfoxide (DMSO) were purchased from Sigma-Aldrich (Darmstadt, Germany). High-glucose Dulbecco’s modified Eagle’s medium (DMEM), fetal bovine serum (FBS), penicillin–streptomycin stock solution (PS, 10,000 U/ml), phosphate-buffered saline (PBS), and pancreatin solution were purchased from Gibco (Carlsbad, CA, USA). The reference standards vicenin-2, luteolin 6-*C*-*β*-d-xyloside-8-*C*-*β*-d-glucoside, vicenin-1, luteolin-6-*C*-*β*-d-glucoside-8-*C*-*β*-d-xyloside, quercetin-3-*O*-sophoroside, schaftoside, luteolin-6-*C*-*β*-d-glucopyranoside, vicenin-3, apigenin-6-*C*-*β*-d-glucoside-8-*C*-*α*-arabinoside, rutin, isoquercitrin, apigenin-6-*C*-*α*-L-arabinoside-8-*C*-*β*-d-xyloside, quercetin 3-*O*-(6″*-O*-malonyl)-*β*-d-glucoside, kaempferol-3-*O*-rutinoside, astragaline, and isorhamnetin-3-*O*-glucoside were purchased from Nature Standard Biotech Co. (Shanghai, China). Aminoguanidine hydrochloride (AG) and glucose were purchased from Aladdin (Shanghai, China), and methylglyoxal (MGO) was purchased from Adamas (Delaware, USA). The Cell Counting Kit-8 (CCK-8), FRAP kit, and SA-*β*-gal staining kit were purchased from Beyotime Biotechnology Co. (Shanghai, China). Alexa Fluor^®^488 Donkey anti-mouse IgG was purchased from Thermo Fisher (Waltham, Massachusetts, USA). Carboxymethyl lysine antibody, Alexa Fluor^®^ 488 donkey anti-mouse IgG, Alexa Fluor^®^ 488 donkey anti-rabbit IgG, Alexa Fluor^®^ 568 goat anti-rabbit IgG, type I collagen primary antibody, type III collagen primary antibody, and mounting medium with 4′,6-diamidino-2-phenylindole (DAPI) were purchased from Abcam (Cambridge, UK). The types I and III collagen enzyme-linked immunosorbent assay (ELISA) kits were purchased from BIO-SWAMP Co. (Wuhan, China). The human dermal fibroblasts (HDFs) were purchased from Archgene Biotechnology Co. (Shanghai, China). Zebrafish were purchased from Hunter Biotechnology Co. (Hangzhou, China). All other chemicals were purchased from Titan Co. (Shanghai, China). Water was purified using a Milli-Q purification system (Barnstead, USA).

### 2.2 Extraction procedure

Aqueous extracts of *D. officinale* flower (DOF) were prepared using slightly modified methods [3]. Briefly, the pulverised flower powder was reflux-extracted twice with distilled water at a solid-to-solvent ratio of 1:12 (w/v) for 1 h at 100°C. The extract was separated through centrifugation at 19,000×*g* for 15 min at 4°C (RWB3220CY-2, Eppendorf, Germany). The supernatant was evaporated at 65°C using a scale rotary evaporator (Hei-VAP Expert, Heidolph; Schwabach, Germany) until a small volume remained, then lyophilised using a freeze dryer. The DOF hot-water extract (DOF-W) was then stored at −18°C until further analysis.

### 2.3 Antioxidant capacity (DPPH·, ABTS·+, and FRAP assays)

Fast colourimetric methods were slightly modified for the *in vitro* assessment of DPPH· scavenging (19), ABTS·+ decolourisation capacity (20), and total antioxidant capacity of FRAP (21). The stock solutions of derivatisation reagents were diluted as follows before measurement: DPPH was diluted with absolute ethanol until the absorbance was 0.8 ± 0.05 at *λ* = 517 nm. ABTS working solution was prepared with phosphate buffer (0.2 M, pH 7.4) to display absorbance of 0.8 ± 0.05 at *λ* = 729 nm, based on a previous study (22). The FRAP solution was prepared according to the instructions of the FRAP kit. Absorbances were measured using an automatic microplate reader (Molecular Group Ltd., USA), and all analyses were performed using 96-well plates. All measurements were performed in triplicate, and Trolox was used as a positive control.

For DPPH· scavenging, 100 μl sample solution was mixed with 100 μl fresh DPPH ethanolic solution, and the absorbance of the mixture was measured after 10 min at 517 nm. Ethanol was used as a negative control.

For ABTS·+ decolourisation capacity, 100 μl of ABTS working solution was mixed with 200 μl of sample solution, and the absorbance was recorded after 10 min at 729 nm. Phosphate buffer (0.2 M, pH 7.4) was used as a negative control.

DPPH· and ABTS·+ radical scavenging activities of the tested sample were both calculated using the following formula:

% inhibition = (*A* control − *A* sample)/A control × 100

Where *A* control and *A* sample represent the absorbance of the control and test samples, respectively. The IC_50_ was calculated graphically from the dose-inhibition curves.

For total antioxidant capacity, 180 μl of FRAP solution was mixed with 5 μl of sample solution, and the absorbance was measured after 5 min at 539 nm with PBS as the negative control. FeSO_4_ solutions (0.15, 0.3, 0.6, 0.9, 1.2, and 1.5 mM) were used for the calibration curve. The FRAP value represents the corresponding concentration of FeSO_4_ solutions (mM FeSO_4_). The regression equation from the standard curve was used to calculate the equivalent concentration 1 (EC_1_) of each sample. EC_1_ is defined as the concentration of the test sample with an absorbance equivalent to that of 1.0 mmol/L of FeSO_4_ solution, determined by its calibration curve (23).

### 2.4 Cellular antioxidant activity assay

#### 2.4.1 Cell culture and UVB treatment

Primary human epidermal keratinocytes (NHEKs) were purchased from Lifeline^®^ Cell Technology (Frederick, MD, USA) and cultured in DermaLife K Keratinocyte Calcium-Free Medium (Cat. LL-0029). NHEKs were incubated in a humidified incubator with 5% CO_2_ at 37°C. NHEKs were exposed to a spectral peak at 312 nm of the UVB irradiation by using an UVB lamp (Spectroline Model EB-160C, New York, NY, USA) at doses of 10 mJ/cm^2^. After UVB irradiation, the cells were washed with warm PBS, and then fresh medium with and without different concentrations of DOF-W (10 and 40 μg/ml) was added and incubated for 24 h.

#### 2.4.2 Cell viability assay and measurement of ROS generation

Cell viability was determined using the CCK-8. After 24 h of incubation, the optical density (OD) was recorded using a microplate reader at 450 nm, according to the instructions of the kit. Based on the methods reported in a previous study [4], the relative levels of ROS were detected using CellROX^®^ Reagent (Life Technologies, Waltham, MA USA). Briefly, NHEKs were seeded in 96-well microplates at 2 × 10^4^ cells/well for 48 h, followed by the UVB treatment and incubation periods specified above. The cells were then incubated with 5 µM CellROX^®^ for 1 h and washed thrice with PBS. ROS production was measured through the fluorescent intensity with the excitation and emission wavelengths set at 485 and 520 nm, respectively.

### 2.5 Anti-COX-2 assay

Anti-inflammatory activity was assessed by measuring COX-2 enzymatic inhibition on the basis of a described method by using the COX-2 Inhibitor Screening Kit (Beyotime, No. S0168) [5]. Celecoxib, a COX-2 inhibitor, was used as a positive control. The results of anti-COX-2 activities are presented as IC_50_ values (μg/ml or μM), a measurement of the inhibition of enzyme activity by each sample by 50%.

### 2.6 Anti-glycation capacity

#### 2.6.1 Fluorescent AGEs formation in a BSA-fructose/glucose system

The formation of total fluorescent AGEs in glycated samples was assessed by determining their fluorescent intensities at an excitation/emission wavelength of 350/450 nm, as previously described (24). Glycated protein was prepared *in vitro* by incubating BSA in the presence of d-glucose (GLC) and d-fructose (FRC). The DOF extract and specific standards were dissolved in DMSO:water (1:4). BSA (4 mg/ml, 100 μl) was preliminary mixed with 50 μl of GLC (0.5 M) and 50 μl of FRC (0.5 M) in 20 mM sodium phosphate buffer (pH 7.4), and then 100 μl samples were added in the 96-well plate and incubated at 37.5°C for 7 days. All solutions were prepared under sterile conditions and filtered using a 0.22-μm syringe before incubation. The assay was performed in triplicate, and AG was used as a positive control. The percentage inhibition of fluorescent AGE formation was calculated using the following equation:


$$\begin{array}{c} Inhibition\;\left(\%\right)\;=\;1\;-\;\\ \left[\left(IF\;sample\;-\;IF\;sample\;control\right)/\left(IF\;control\;-\;IF\;blank\;control\right)\right]\;\times \;100\%. \end{array}$$

Where IF sample is fluorescence intensity in the presence of samples and BSA, IF sample control is fluorescence intensity in the presence of samples without BSA, IF control is fluorescence intensity without samples, and IF blank control is fluorescence intensity without samples and BSA. The results of anti-glycation activities are presented as IC_50_ values (μg/ml or μM), a measurement of the 50% inhibition of enzyme activity by each sample.

#### 2.6.2 Glycation cell assay

Based on previously reported methods (25), human primary dermal fibroblasts (HDFs) were induced by MGO to establish a cell model of high CML expression.

HDFs were cultured in high-glucose DMEM supplemented with 10% FBS containing 100 g/ml of PS at 37°C in a humidified 5% CO_2_ incubator. Cell viability was assessed using the CCK-8 kit. HDFs were cultured in 12-well plates to approximately 70% confluence and then treated with 0.5 mmol/L of MGO for 48 h to induce glycation and the formation of CML. Subsequently, different concentrations of samples were added and co-cultured with MGO for an additional 48 h. Cells were then washed once with PBS and fixed in polyformaldehyde overnight at 4°C. Triton-X-100 solution (0.1%) was assigned to fixed cells for 5 min at room temperature to improve cell membrane permeability. After washing twice with PBS, cells were blocked in 1% BSA for 1 h, followed by incubation with the primary CML antibody (1:50) overnight at 4°C. The secondary antibody donkey-anti-Mouse Alexa Fluor^®^ 488 (1:1,000) was then added for 2 h at room temperature. Counterstaining was performed by adopting anti-fluorescence quenching and sealing liquid containing DAPI, followed by visualisation and fluorescence image capture using a microscope (Leica, Germany). ImageJ software was used to quantify CML expression from different images randomly selected in each group.

### 2.7 Anti-aging assay on HDFs

#### 2.7.1 Cell culture

HDFs were cultured in DMEM with 10% (v/v) FBS and 1% PS at 37°C in a humidified 5% CO_2_ incubator. Cells were subcultured using pancreatin solution after reaching confluence. All experiments were performed between the third and eighth passages.

#### 2.7.2 Quantification of collagen types I and III using immunofluorescence

The synthesis of collagen types I and III was detected using IF (26) and ELISA (27). HDFs were digested and seeded in a 24-well culture plate on slides at a density of 50,000 cells/well in a complete medium. After reaching 80% confluency, cells were starved with the medium in the absence of serum for 16 h. Cells were then treated with positive controls or active samples (prepared in medium without serum) for an additional 48 h. For analysis and visualisation of types I and III collagens, the supernatants and remaining cell slides were both collected and analysed using ELISA kits and the IF staining method, respectively.

Collected supernatants were centrifuged at 10,000 rpm for 10 min and used for types I and III collagen ELISA, according to the manufacturer’s protocol. The remaining cell slides were washed once with PBS and fixed in polyformaldehyde overnight at 4°C. Subsequently, the slides were treated with 0.5% Triton-X100 for 5 min. After washing, cells were blocked with 1% BSA at room temperature for 1 h. Cells were then incubated with types I and type III collagen primary antibodies (1:100) for 2 h at room temperature. After washing, cells were stained with secondary antibody (1:1,000) for 1 h (Alexa Fluor^®^ 568 goat anti-rabbit IgG and Alexa Fluor^®^ 488 donkey anti-rabbit IgG were used to detecting fluorescence of types I and type III collagen, respectively). Finally, the nuclei were counter-stained with DAPI, and relevant fluorescent images were captured using a microscope. ImageJ software was used to quantify collagen expression from different images randomly selected in each group.

#### 2.7.3 SA-*β*-gal staining assay

Senescent human skin fibroblasts induced by d-galactose (d-gal) were established based on a previously reported method (28, 29). HDFs were divided into the control, aging model, and sample (aging model with DOF extract) groups. Except for the control group, HDFs were cultured in six-well plates to approximately 70% confluence and then treated with 20 mg/ml d-gal for 72 h to induce cell senescence. Subsequently, different concentrations of DOF extract were added to the sample group and co-cultured with d-gal for an additional 72 h. The media was then aspirated from cells, and the wells were washed once with PBS. SA-*β*-gal staining was performed using an SA-*β*-gal staining kit (Beyotime Co.). Fixative was added and incubated for 10 min at room temperature. Cells were then washed twice with PBS and incubated in 1 ml of SA-β-gal staining solution (freshly prepared according to the protocol of the kit). Plates were maintained in the dark, overnight, in a humidified incubator at 37°C without CO_2_. The following day, the staining solution was removed and cells were maintained in the final solution of PBS. The staining of SA-*β*-gal was observed, and relevant images were captured using a microscope. ImageJ software was used to determine the average value of the proportion of blue-stained cells of five randomly selected microscopic images in each group.

### 2.8 UPLC-PDA-QTOF-ESI-MS/MS analysis

DOF extract (1 mg) was diluted with 1 ml of distilled water, sonicated for 20 min, and filtered using a 0.45-μm syringe filter before analysis.

The UPLC analysis was performed using an Agilent 1290 UPLC system (California, USA) combined with an Agilent Q-TOF 6545 LC/MS system, a sample manager, a PDA detector, and a binary solvent manager, and was controlled using MassHunter Workstation Software. The Acquity HSS T3 reverse phase column (2.1 × 100 mm, 1.8 μm; Waters, Milford, MA, USA) at a separation temperature of 30°C was used to perform the chromatographic separation of 2 μl of each sample with a wavelength scanning range of 190–400 nm. Gradient elution at a flow rate of 0.2 ml/min was completed with the mobile phase consisting of solvent A (0.2% acetic acid in ultrapure water) and solvent B (acetonitrile) in the following order: 0–5 min, 2% B; 5–8 min, 2%–10% B; 8–12 min, 10% B; 12–20 min, 10%–15% B; 20–28 min, 15%–20% B; 28–31 min, 20% B; 31–38 min, 20–80% B; and 38–40 min, 80% B. Finally, the initial conditions were reintroduced over the course of 2 min. Before each run, the column was equilibrated for an additional 2 min. The MS was operated in both positive and negative ion modes. The optimised MS conditions were as follows: TOF mass range, m/z 50–1,700; ion source gas, 50 psi; curtain gas, 35 psi; ion spray voltage, 5 kV; ion source temperature, 500°C; and collision energy, 10 eV. The following MS/MS parameters were applied: MS/MS mass range, 50–1,250 m/z; collision energy, 40 eV; declustering potential, 100 V; and collision energy spread, 20 eV. Compounds were identified and analysed by comparing their retention times, fragment ions, and formulas using corresponding standards and commercial databases.

### 2.9 Antioxidant online profiling using UPLC-PDA-QDa coupled with postcolumn derivatisation with ABTS

Online identification of antioxidant components of DOF extract was performed using a UPLC system (Milford, MA, USA) consisting of a Waters photodiode array detector and a Waters postcolumn derivatisation system supplying fresh ABTS solution (UPLC-PDA-QDa-ABTS). Gradient elution at a flow rate of 0.8 ml/min was completed with the mobile phase consisting of solvent A (0.2% acetic acid in ultrapure water) and solvent B (acetonitrile) in the following order: 0–5 min, 2% B; 5–8 min, 2%–10% B; 8–12 min, 10% B; 12–20 min, 10%–15% B; 20–28 min, 15%–20% B; 28–31 min, 20% B; 31–38 min, 20%–80% B; and 38–40 min, 80% B. Separation of compounds was performed at 30°C using an Acquity reversed-phase column (4.6 × 250 mm, 5 μm; Waters, Milford, MA, USA). The detection wavelengths were set at 280 and 734 nm, and the injection volume of the sample was 10 μl.

**Figure 1:**
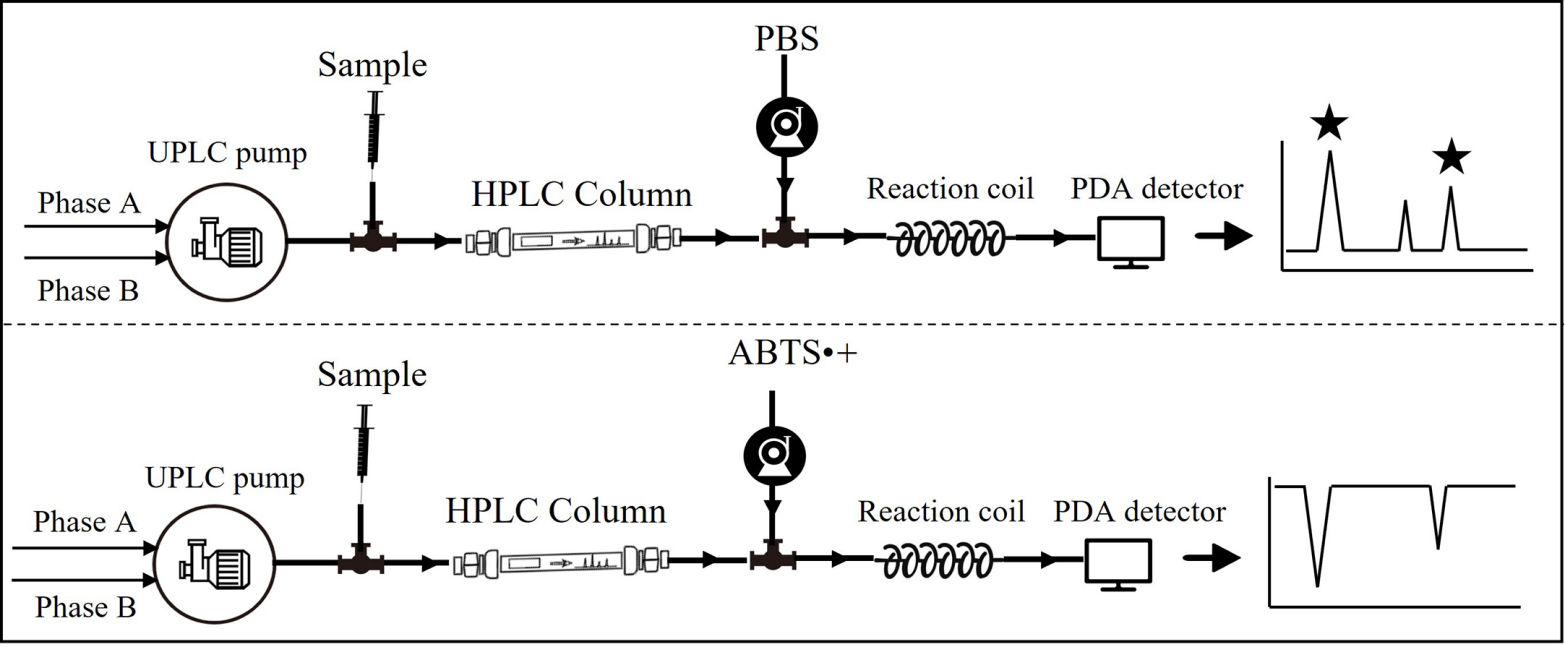
Flow chart of online detection of antioxidants.

The technical route and relevant device installation are shown in **Figure 1**. To detect radical scavengers, the UPLC system was coupled with a Waters pump, which supplied freshly prepared ABTS·+ solution into a reaction coil (15 m, 0.25 mm ID) with a flow rate of 0.2 ml/min at 37°C. Negative peaks were recorded based on a decrease of absorbance at 729 nm after the reaction of individual compounds with the ABTS·+ radical. For preliminary identification of compounds, a Waters Acquity QDa mass detector in negative ionisation mode was connected in series to PDA with the following parameters: electrospray ion source; cone voltage, 15 V; atomiser, N2; and scanning range, 100–800 m/z.

### 2.10 Statistical analysis

The data were statistically processed using GraphPad software. The test data were expressed as mean ± standard deviation (SD), and significance was evaluated by one-way analysis of variance (ANOVA) and Tukey’s test. Statistical significance was indicated as follows: ^*^ *p*< 0.05 and ^**^ *p*< 0.01.

## 3 Results

### 3.1 *In vitro* antioxidant potency of DOF extracts

The growing awareness of herbs with antioxidant properties has been noted over the last few decades, mainly due to the discovery of ROS closely involved in chronic non-infectious diseases. Using several antioxidation assays and various models is vital for a more comprehensive assessment of natural products. Therefore, in the present study, DPPH, ABTS, and FRAP assays, as well as intracellular ROS levels induced by UVB radiation in NHEKs cells, were used for measuring the antioxidant potential of the *D. officinale* flower aqueous extract (DOF-W).

**Figure 2:**
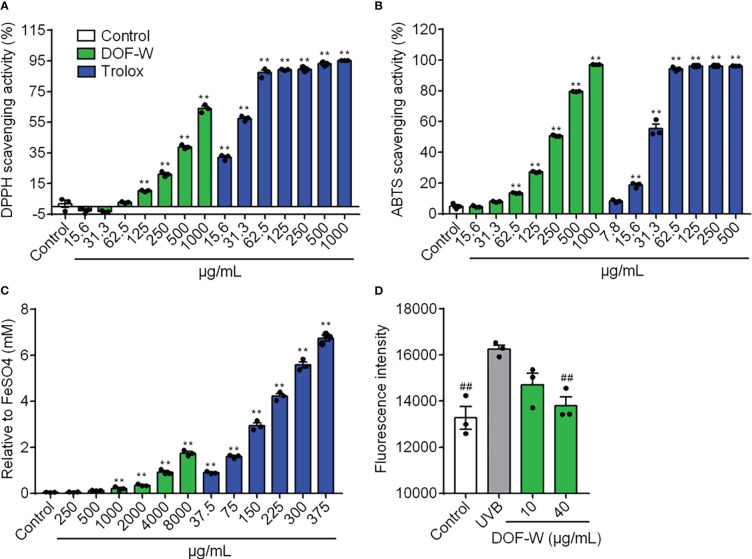
Antioxidant potency of DOF extracts. DPPH scavenging activity **(A)**, ABTS scavenging activity **(B)**, FRAP value **(C)**, and intracellular ROS level **(D)** of DOF-W. Compared with the control, ^**^ *p<* 0.01. Compared with the UVB model, ^##^ *p<* 0.01.

For the concentration range of 15.6–1,000 μg/ml, DOF-W showed antioxidant activity in a dosage-dependent manner, as shown in **Figure 2**. For the DPPH and ABTS assays, the IC_50_ value of DOF-W was 669.7 ± 20.59 μg/ml and 224.57 ± 0.65 μg/ml, respectively, whereas the IC_50_ values of Trolox were 24.7 ± 1.0 μg/ml and 27.68 ± 1.04 μg/ml. For the FRAP assay, the EC1 value of DOF-W was 4,580 ± 260 μg/ml, whereas the EC1 value of Trolox was 55.35 ± 5.22 μg/ml. Though DOF-W showed a weaker antioxidant ability compared to a classical antioxidant standard (Trolox), it still exhibited potential antioxidant activity, particularly at high concentrations, which were consistent with various previous studies (30, 31).

Safety and non-toxicity are essential for functional foods, as well as skin-care cosmetics. The DOF-W had no significant damage to NHEK cell viability in the range of 1–40 μg/ml (**Supplementary Data**). After being irradiated by UVB (10 mJ/cm^2^), cell survival was significantly reduced. However, DOF-W did not further aggravate UVB-induced NHEK cell mortality (**Supplementary Data**). As **Figure 2D** shows, UVB irradiation leads to an increasing ROS level of NHEK cells. Treatment with 40 μg/ml of DOF-W notably reduced intracellular ROS levels.

### 3.2 Anti-COX-2 capacity of DOF extracts

As COX-2 is an inducible enzyme that produces prostaglandins (PGs) and is responsible for generating ROS, it is always regarded as a pathologic enzyme chiefly responsible for inflammation (32), and COX-2 inhibitors, which can cause a sharp drop in the amount of ROS, are also found to be highly associated with potential antioxidant effects (33). In this study, the COX-2 inhibition assay was used to evaluate the anti-inflammatory effect of DOF-W.

**Figure 3:**
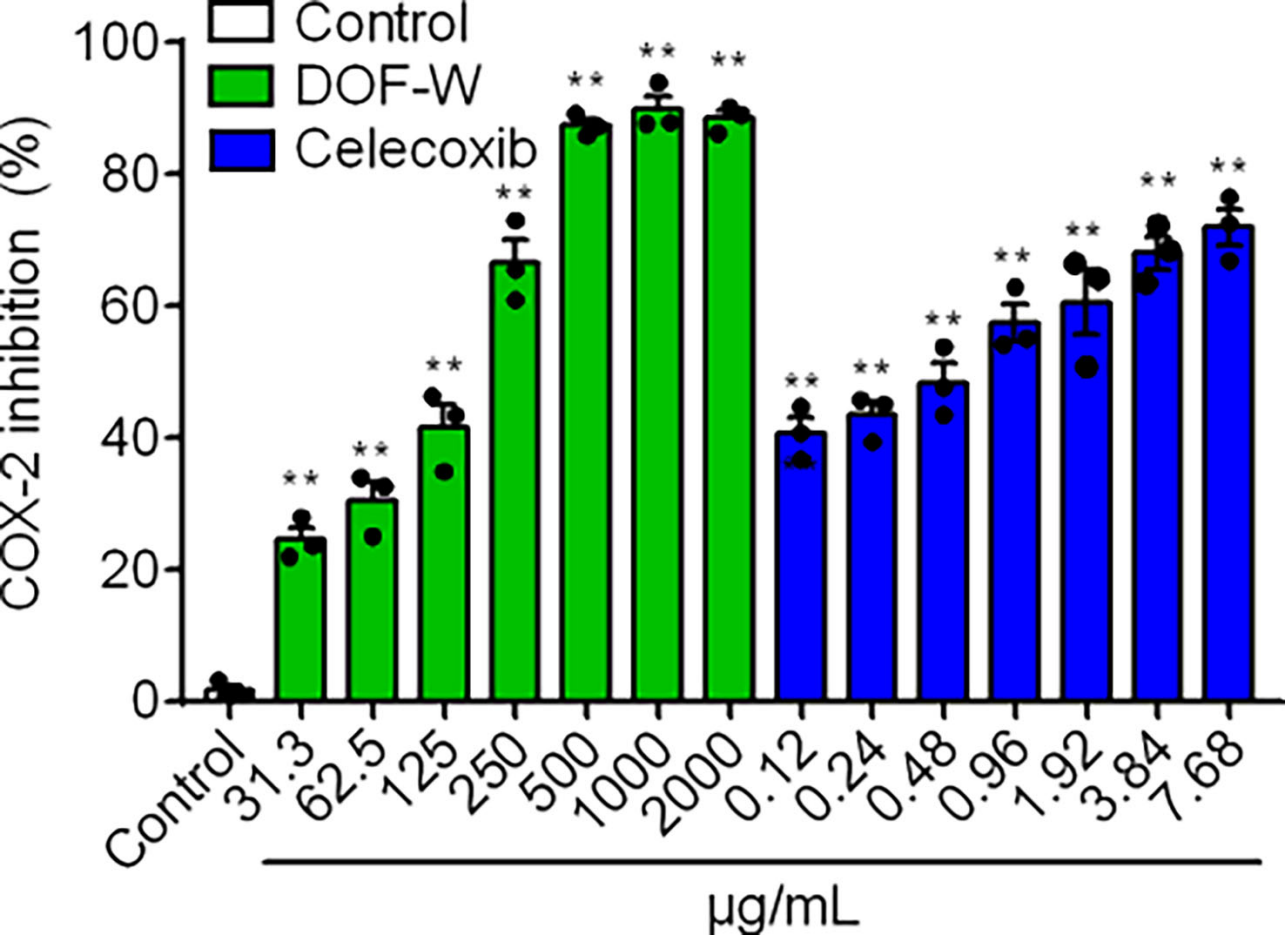
COX-2 inhibition of DOF-W. Compared with the control, ^**^ *p<* 0.01.

The COX-2 inhibitory activity of DOF-W was compared with a well-known selective COX-2 inhibitor (Celecoxib). For the concentration range of 31.3–2,000 μg/ml, DOF-W showed great concentration-dependent COX-2 inhibitory activity, as shown in **Figure 3**. The DOF-W had an IC_50_ value of 133.5 ± 27.4 μg/ml, whereas Celecoxib had an IC_50_ value of 0.53 ± 0.30 μg/ml. Although DOF-W may show lower capacities due to the dilution of the active constituents with neutral ones, it still could be used as a promising COX-2 inhibitor.

### 3.3 Anti-glycation activity of DOF-W

Glycosylation within tissues is a slow and complicated process. Excessive generation and accumulation of AGEs in the process will cause irreversible damage to the body. Various AGEs in skin collagen, including CML, can damage skin fibroblasts, resulting in skin aging and the formation of dark spots (10). In the present study, the determination of anti-glycation activities was evaluated by multimodal methods, including routine chemical tests, cell experiments, and a zebrafish assay.

#### 3.3.1 Inhibition of DOF-W on total fluorescent AGEs of non-enzymatic glycation

**Figure 4:**
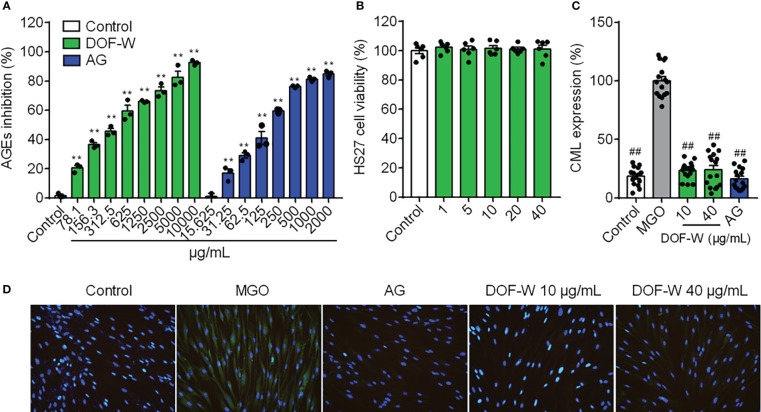
Anti-glycation activity of DOF-W. **(A)** Inhibition of fluorescent AGEs by DOF-W extract. **(B)** Cytotoxicity of DOF-W extract to fibroblasts. **(C)** Effect of CML expression in glyoxal-induced HDFs by DOF-W extract. **(D)** Representative images of HDFs by fluorescence microscope and statistics. ^##^ *p<* 0.01 compared with the MGO model. ^**^ *p<* 0.01 compared with control. AG, aminoguanidine hydrochloride; MGO, methylglyoxal; CML, *N*-carboxymethyl lysine.

Incubation of reducing sugars and BSA induced the production of fluorescent AGEs (34). In this study, the BSA-fructose/glucose as a model reaction system was first applied to preliminarily evaluate the effect of DOF aqueous extracts on the inhibition of AGEs. As shown in **Figure 4A**, DOF-W extracts can reduce levels of fluorescent AGEs in a concentration-dependent relationship with IC_50_ values of 428.4 ± 75.1 μg/ml. AG, a synthetic AGE inhibitor, expectedly displayed an effective inhibition with IC_50_ values of 177.3 ± 11.6 μg/ml.

#### 3.3.2 Anti-glycation effect of DOF-W in human primary fibroblasts

Anti-glycation primary assay of BSA/reducing sugar system as the above showed that DOF extract was a potential anti-glycans. The DOF extract was then further tested in an *in vitro* glycation assay to confirm its activities on cells. MGO is a critical and potent precursor in the formation of AGEs, reacting with proteins to produce *N*ϵ-carboxymethyl lysine (CML), which is one of the principal AGEs in the skin without fluorescence properties and cannot be detected by conventional assay (35). Fibroblasts were treated with MGO to induce glycation, which was then visualised and quantified using IF. No obvious CML green fluorescence staining in the blank control group was observed, which indicated that normal cells hardly secrete CML, whereas a large amount of CML was expressed in MGO-induced cells after CML IF, as shown in **Figure 4D**. The treatment of the cells with the positive compound AG resulted in significant inhibition of glycation by 88.11%. Since no cytotoxicity was found under concentrations of 40 μg/ml, as shown in **Figure 4B**, treatment with 10 and 40 μg/ml of DOF extract in HDFs both resulted in significant inhibition of glycation by 77.88% and 69.70%, respectively, without a distinct dose-dependent manner (**Figures 4C, D**).

### 3.4 Effect of DOF extracts on collagen synthesis in human skin fibroblast cells

Dermal fibroblasts are thought to be responsible for synthesizing various dermal ECM proteins, including fibrous collagens. Type I collagen accounted for approximately 80% of total collagen, whereas type III collagen is more prevalent in young skin than aged skin and is particularly involved in wound healing (36). Since skin aging is characterized by the degradation of ECM components such as types I and III collagen breakdown, we investigated whether DOF could enhance the expression of types I and III collagen in HDFs.

**Figure 5:**
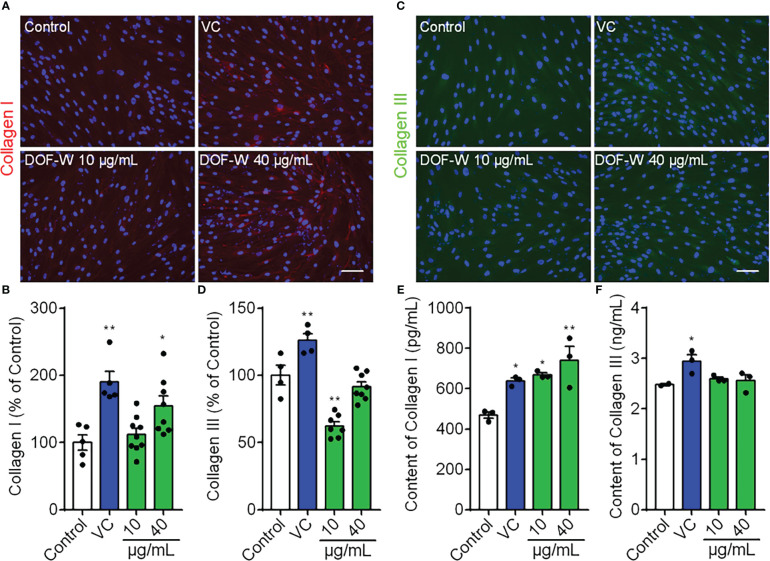
Effects of DOF extract on the expression of collagens by IF staining. HDFs were treated with VC (200 μg/ml, positive control) and DOF extract (10 and 40 μg/ml), whereas HDFs were incubated without any treatment as the control group. **(A)** Images of collagen I staining (red color) and quantification **(B)**. **(C)** Images of collagen III staining (green color) and quantification **(D)**. Extracellular content of types I **(E)** and III **(F)** collagen on HDFs using ELISA. Bar scale, 100 μm. Compared with the control group, ^*^ *p*< 0.05, ^**^ *p*< 0.01.

As shown in **Figures 5A, B**, IF assay revealed that DOF-treated cells synthesized more amount of type I collagen than untreated control cells after 48 h. DOF increased the extent of collagen type I staining by 111.88% and 154.59% of control at a concentration of 10 and 40 μg/ml, respectively. However, the level of expression of type III collagen was not statistically altered or even decreased by DOF extract, as shown in **Figures 5C, D**. Collagen types I and III expressions were significantly increased by vitamin C by 190.20% and 125.90% of control as a positive drug, respectively.

We also tested the extracellular content of collagen types I and III for 48 h on HDFs in the culture medium. As shown in **Figures 5E, F**, treatment with vitamin C (VC), 10 μg/ml of DOF, and 40 μg/ml of DOF increased secreted collagen type I levels by 21.94%, 42.6%, and 57.65%, respectively, whereas DOF had no obvious effect on type III collagen secretion, further confirming the corresponding significantly stronger effect on collagen type I. Therefore, we conclude that DOF extracts can upregulate collagen type I level in HDFs, but they have no positive effect on type III collagen.

### 3.5 SA-β-Gal staining assay on DOF-W

Cellular senescence is an irreversible physiological phenomenon in which normal cells have lost their proliferative potential but are still alive and maintaining their metabolic activity (37). Cell senescence can be measured using SA-*β*-gal staining assay, which is widely used to locate SA-*β*-gal-positive cells (blue-stained cells) by optical microscope. Among several existing types of aging models, such as X-ray, H_2_O_2_, and d-Gal-induced aging models, d-gal induced aging models to resemble natural aging but take a shorter time than the latter (38).

**Figure 6:**
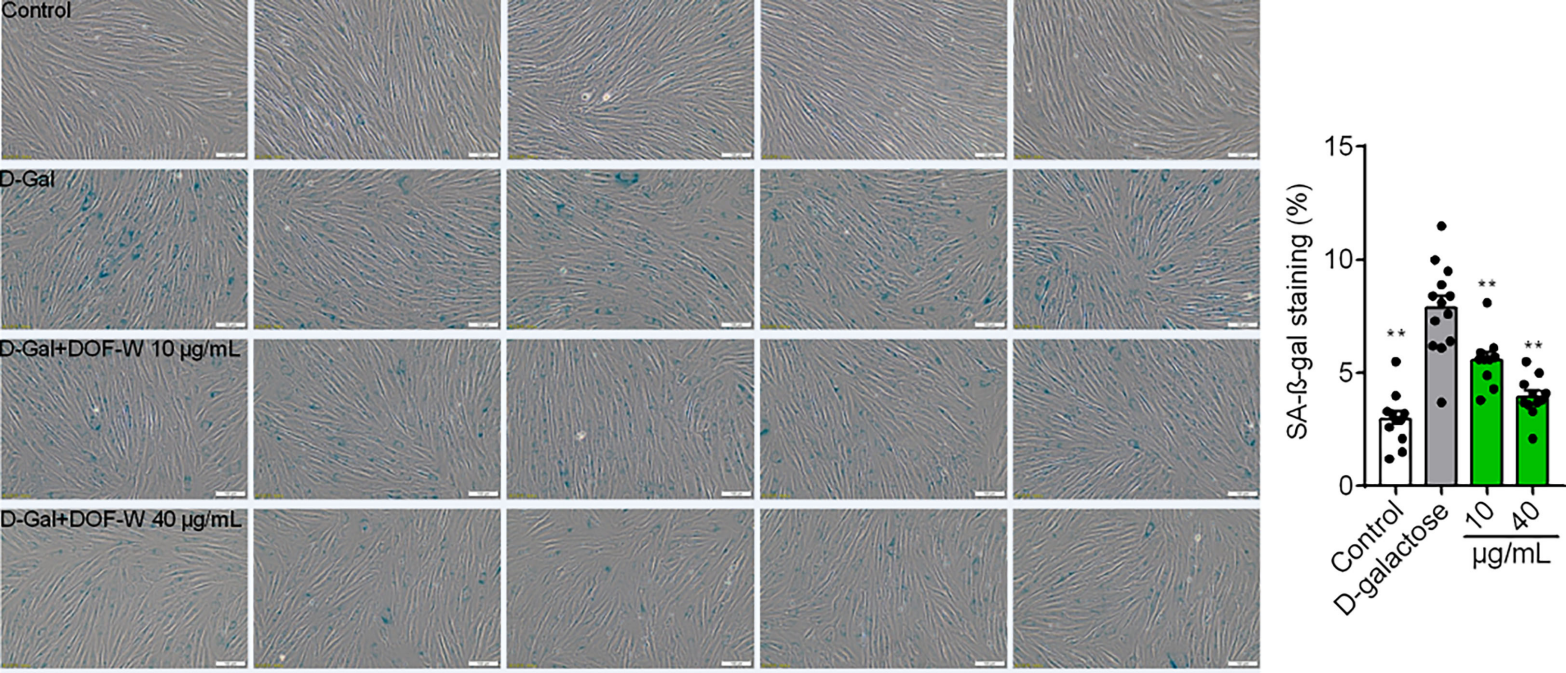
Images of fibroblasts with SA-β-gal staining. Statistics results of fibroblasts with SA-β-gal staining. Bar scale, 100 μm. Compared with the model group, ^**^ *p<* 0.01.

As shown in **Figure 6**, few blue-stained cells were observed in the control group (3.0% ± 1.2%). SA-*β*-gal-positive cells significantly increased after treatment with 20 mg/ml of d-gal for 6 days, consecutively, in the model group (7.9% ± 2.0%). When senescent cells coincubated with 10 and 40 μg/ml of DOF extracts, the proportion of SA-β-gal-positive cells were 4.0% ± 0.9% and 5.6% ± 1.2%, respectively, which were conspicuously lower than those of the model group.

### 3.6 Determination of phytochemicals using UPLC-Q/TOF-MS/MS

**Figure 7:**
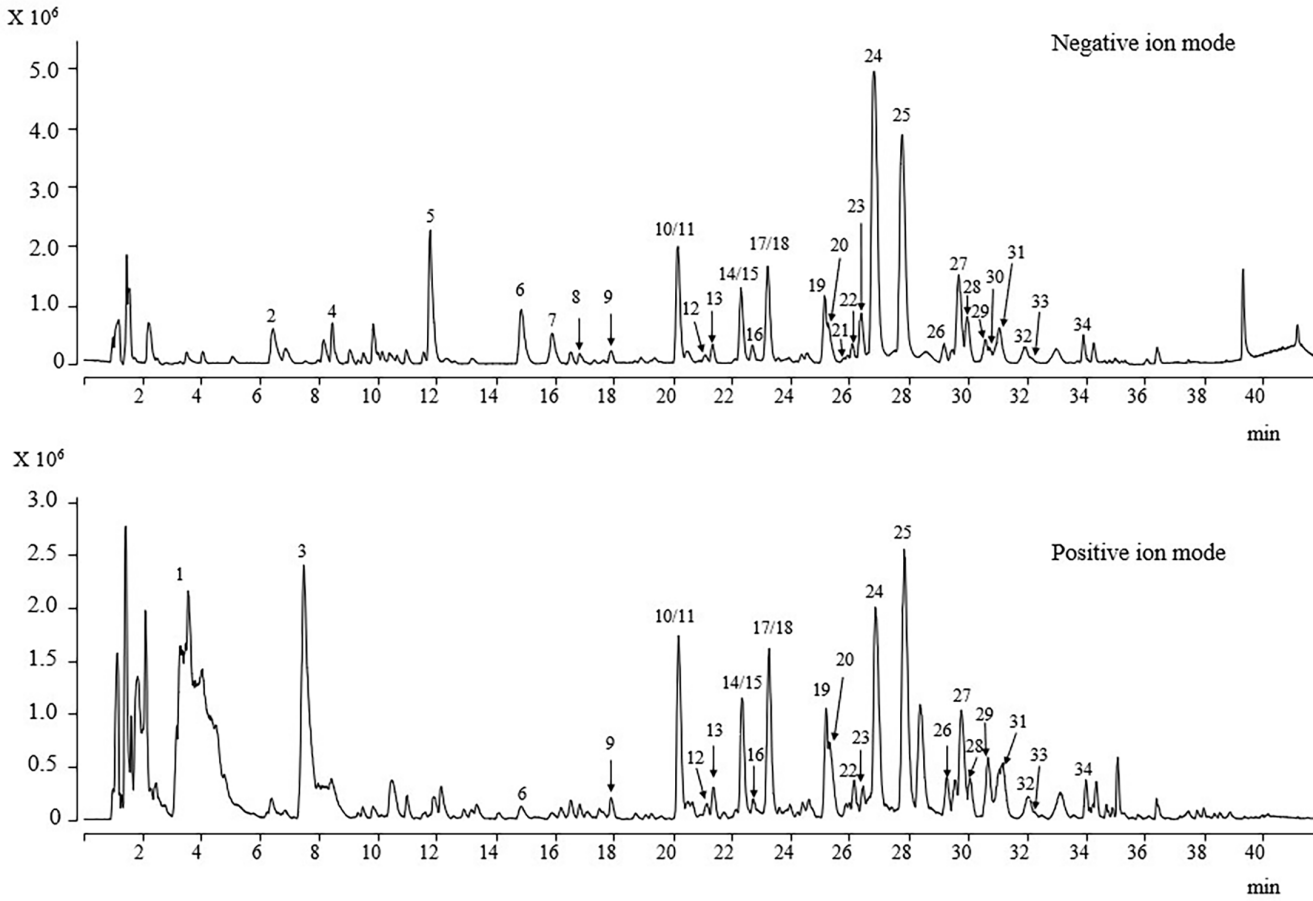
UPLC-ESI/MS/MS total ion chromatogram of DOF-W.

**Figure 8:**
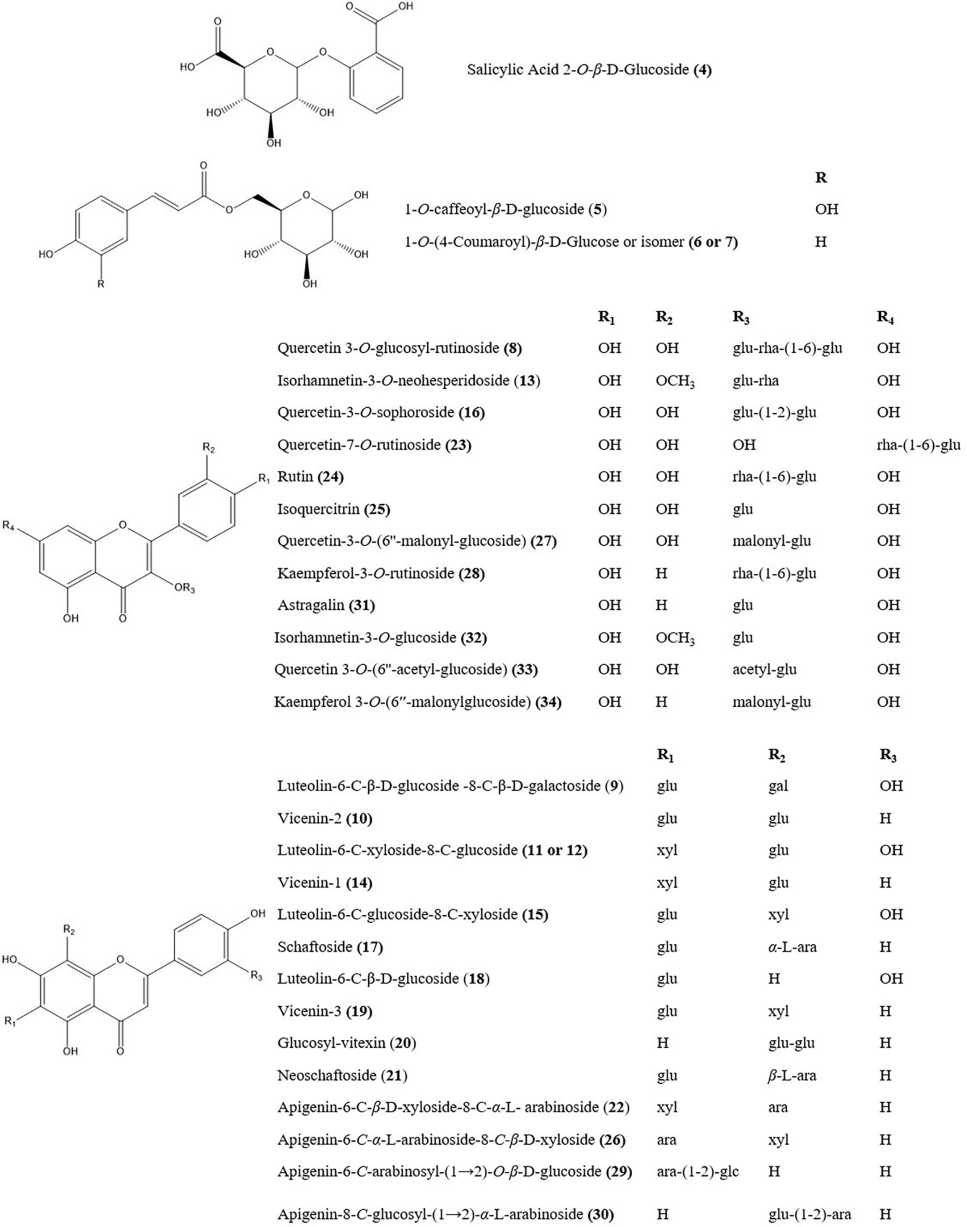
The chemical structures of main herb markers in the DOF-W.

The 25 compounds were further characterised by structural analysis using UPLC-PDA-ESI-Q/TOF-MS/MS (**Table 1**; **Supplementary Materials**). The total ion chromatogram of the aqueous extract of the sample under both negative and positive ion mode is shown in **Figure 7**. The retention time (Rt), molecular formula, and ion and ions after fragmentation are shown in **Table 1**. The Rt and fragmentation information of compounds 13, 14, 16, 17, 19, 20, 23, 24, and 25 were compared with those of standards. The major constituents of *DOF-W* were flavonoids (**Figure 8**). A typical MS spectrogram fragmentation mechanism for flavonoid disaccharide C-glycoside such as schaftoside is shown in **Supplementary Figure S2**.

**Table 1:** The physical properties of the identified compounds from the flowers of *Dendrobium officinale*.

No.	*T*_(min)_	Ion	*m/z*	ppm	Formula	Mol. wt.	Name	MS/MS data
1	3.08	[M+H]^+^	294.1547	−2.0	C_12_H_23_NO_7_	293.15	Fructoseleucine	276.1445; 258.1295; 230.1362; 212.1264; 182.1158
2	5.78	[M−H]^−^	282.0842	1.3	C_10_H_13_N_5_O_5_	283.09	Guanosine	150.0397; 133.0150; 108.0204; 78.9594
3	6.83	[M+H]^+^	268.1047	0.5	C_10_H_13_N_5_O_4_	267.10	Adenosine	136.0618; 119.0350; 94.0394; 57.0330
4	8.04	[M−H]^−^	299.0776	3.0	C_13_H_16_O_8_	300.08	Salicylic acid 2-*O*-*β*-d-glucoside	137.0238; 123.0080; 93.0346
5	11.49	[M−H]^−^	341.0889	4.8	C_15_H_18_O_9_	342.10	1-*O*-caffeoyl-*β*-d-glucoside	179.0347; 161.0246; 135.0448; 133.0296
6	14.41	[M−H]^−^	325.0935	3.6	C_15_H_18_O_8_	326.10	1-*O*-(4-coumaroyl)-*β*-d-glucose or isomer	163.0392; 145.0293; 117.0345; 59.0135
7	15.45	[M−H]^−^	325.0935	3.6	C_15_H_18_O_8_	326.10	1-*O*-(4-coumaroyl)-*β*-d-glucose or isomer	163.0376; 145.0296; 117.0348; 59.0139
8	16.53	[M−H]^−^	771.1987	0.4	C_33_H_40_O_21_	772.21	Quercetin 3-*O*-glucosyl-rutinoside	771.1974; 609.1456; 463.0794; 301.0343; 178.9978
9	17.57	[M−H]^−^	609.1461	0.2	C_27_H_30_O_16_	610.15	Luteolin-6-*C-β*-d-glucoside-8-*C-β*-d-galactoside	489.1025; 429.0796; 399.0700; 369.0604
10	19.86	[M−H]^−^	593.1519	2.1	C_27_H_30_O_15_	594.16	Vicenin-2	593.1533; 473.1087; 383.0768; 353.0659; 297.0749
11	19.86	[M−H]^−^	579.1352	1.3	C_26_H_28_O_15_	580.14	Luteolin- 6-*C-β*-d-xyloside-8*-C-β*-d-glucoside	429.0776; 399.0709; 369.0602; 339.0487
12	20.82	[M−H]^−^	579.1355	−0.2	C_26_H_28_O_15_	580.14	Luteolin-6-*C*-xyloside-8-*C*-glucoside isomer	459.0916; 429.0811; 399.0711; 369.0605; 339.0518
13	21.06	[M−H]^−^	623.1620	0.0	C_28_H_32_O_16_	624.16	Isorhamnetin-3-*O*-neohesperidoside	503.1182; 413.0874; 383.0774; 357.0621; 315.0635
14	22.03	[M−H]^−^	563.1398	−0.5	C_26_H_28_O_14_	564.15	Vicenin-1	563.1490; 503.1179; 473.1081; 383.0680; 353.0667
15	22.03	[M−H]^−^	579.1353	−0.2	C_26_H_28_O_15_	580.14	Luteolin-6-*C*-β-d-glucoside-8*-C-β*-d-xyloside	429.0799; 399.0710; 369.0616
16	22.36	[M−H]^−^	625.1396	−1.4	C_27_H_30_O_17_	626.15	Quercetin-3-*O*-sophoroside	625.1370; 463.0864; 301.0338
17	22.93	[M−H]^−^	563.1404	0.6	C_26_H_28_O_14_	564.15	Schaftoside	563.1398; 503.1182; 473.1082; 383.0770; 353.0664
18	23.07	[M−H]^−^	447.0932	−0.2	C_21_H_20_O_11_	448.10	Luteolin-6-*C-β*-d-glucoside	411.0716; 357.0613; 327.0495; 298.0475; 285.0412
19	24.91	[M−H]^−^	563.1426	4.5	C_26_H_28_O_14_	564.15	Vicenin-3	563.1391; 473.1082; 443.0974; 383.0768; 353.0663
20	25.14	[M−H]^−^	593.1521	1.0	C_27_H_30_O_15_	594.16	Glucosyl-vitexin	431.0946; 311.0549; 293.0463; 59.0139
21	25.56	[M−H]^−^	563.1404	−0.2	C_26_H_28_O_14_	564.15	Neoschaftoside	473.1073; 444.1005; 413.0873; 383.0770; 353.0649; 311.0548
22	25.87	[M−H]^−^	533.1309	1.1	C_25_H_26_O_13_	534.14	Apigenin-6-*C-β*-d-xyloside-8-*C-α*-l-arabinoside-	413.0855; 383.0770; 353.0660
23	26.07	[M−H]^−^	609.1469	2.2	C_27_H_30_O_16_	610.15	Quercetin-7-*O*-rutinoside	609.1433; 463.0865; 301.0333; 300.0277; 271.0242
24	26.52	[M−H]^−^	609.1484	4.7	C_27_H_30_O_16_	610.15	Rutin	609.1455; 301.0346; 300.0283; 178.9980
25	27.46	[M−H]^−^	463.0897	4.4	C_21_H_20_O_12_	464.10	Isoquercitrin	301.0281; 300.0281; 271.0254; 255.0302; 178.9985
26	29.05	[M+H]^+^	535.1457	1.0	C_25_H_26_O_13_	534.14	Apigenin-6*-C-α*-l-arabinoside-8-*C-β*-d-xyloside	463.0998; 433.0909; 403.0807; 391.0804; 379.0806; 325.0697; 307.0595
27	29.38	[M−H]^−^	549.0882	0.2	C_24_H_22_O_15_	550.10	Quercetin 3-*O*-(6″-malonyl-glucoside)	549.0832; 505.0937; 463.1016; 301.0437; 300.0274; 271.0244; 255.0295
28	29.76	[M−H]^−^	593.1517	1.7	C_27_H_30_O_15_	594.16	Kaempferol-3-*O*-rutinoside	593.1506; 285.0398; 284.0323; 255.0293; 151.0030
29	30.45	[M+H]^+^	565.1561	0.7	C_26_H_28_O_14_	564.15	Apigenin-6-*C*-arabinosyl-(1→2)-*O-β*-d-glucoside	367.0808; 349.0704; 337.0704; 313.0702; 283.0598
30	30.82	[M+H]^+^	565.1538	−3.4	C_26_H_28_O_14_	564.15	Apigenin-8-*C*-glucosyl-(1→2)-*α-* l-arabinoside	367.0810; 349.0697; 337.0704; 313.0702; 283.0601
31	30.83	[M−H]^−^	447.0942	3.3	C_21_H_20_O_11_	448.10	Astragalin	284.0320; 255.0293; 227.0343; 151.0034
32	31.60	[M−H]^−^	477.1038	−0.3	C_22_H_22_O_12_	478.11	Isorhamnetin-3-*O*-glucoside	314.0426; 299.0176; 285.0401; 271.0237; 257.0448; 243.0291
33	31.86	[M−H]^−^	505.0987	−0.1	C_23_H_22_O_13_	506.11	Quercetin 3-*O*-(6″-acetyl-glucoside)	300.0273; 271.0243; 225.0307
34	33.76	[M−H]^−^	533.0935	−0.3	C_24_H_22_O_14_	534.10	Kaempferol 3-*O*-(6″-malonylglucoside)	284.0326; 255.0299; 229.051

### 3.7 Determination of antioxidants by the online UPLC-PDA-MS-ABTS·+ scavenging

To preliminarily screen out antioxidant phytochemicals in DOF-W, in the present study, UPLC coupled with PDA-Qda and ABTS-based assay was performed. The constituents, for which MS data and reference compounds were available, can be identified from positive peaks in **Supplementary Figure S2**. The area of reordered negative peaks on the lower chromatogram at 734 nm conforms to the ABTS radical scavenging activity of individual compounds of the DOF extract. As it may be judged from the size of the negative peaks in the chromatogram, 1-*O*-caffeoyl-*β*-d-glucoside (compound 5), vicenin-2 (compound 10), luteolin-6-*C*-*β*-d-xyloside-8-*C*-*β*-d-glucoside (compound 11), quercetin-3-*O*-sophoroside (compound 16, rutin (compound 24), isoquercitrin (compound 25), and quercetin 3-*O*-(6″-*O*-malonyl)-*β*-d-glucoside (compound 27) were the strongest ABTS radical scavengers in the investigated extract. Since components 10 and 11 are not well separated, it is a challenge to distinguish which component is mainly responsible for the negative peak and needs further confirmation by extra experiments. In a previous report (30), by employing precolumn DPPH and ABTS assay followed by HPLC-DAD analysis, 1-*O*-caffeoyl-*β*-d-glucoside, rutin, and isoquercitrin were identified as major components with obvious scavenging free radical abilities in methanol extract of DOF.

### 3.8 *In vitro* pro-health potency of specific compounds from DOF extracts

In consideration of the time-consuming process of isolating components in flowers and to further identify and confirm the biological activities of components that may contribute to the antioxidative, anti-inflammatory, and anti-glycation properties of the extract, specific available compounds were selected for more detailed investigation on the assays of DPPH, ABTS, FRAP, COX-2, and BSA/reducing sugar glycation. The results of these isolated compounds are shown in **Table 2**.

**Table 2:** Antioxidant (DPPH, ABTS, FRAP), anti-cyclooxygenase (COX-2), and AGE inhibition activity (BSA/reducing sugar reaction) (% of inhibition) of various compounds from *Dendrobium officinale* flower-aqueous extract.

No.	Compound name	DPPH	ABTS	FRAP	COX-2	BSA-glycation
		IC_50_ (μM)	IC_50_ (μM)	EC_1_ (μM)	IC_50_ (μM)	IC_50_ (μM)
10	Vicenin-2 (apigenin 6,8-C-diglucoside)	>5,000	74.41 ± 0.69	>5,000	nd	257.67 ± 7.56
11	Luteolin 6-*C*-*β*-d-xyloside-8-*C-β*-d-glucoside	226.93 ± 47.26	15.90 ± 0.19	1,009.1 ± 143.3	14.2 ± 3.4	251.6 ± 5.1
14	Vicenin-1 (apigenin 6-*C-β*-d-xyloside-8-*C*-glucoside)	>5,000	96.39 ± 2.11	>5,000	nd	255.63 ± 5.44
15	Luteolin-6-*C-β*-d-glucoside-8-*C-β*-d-xyloside	145.13 ± 51.37	13.80 ± 0.82	1,100.3 ± 138.4	13.9 ± 2.4	293.6 ± 13.3
16	Quercetin-3-*O*-sophoroside	172.13 ± 76.03	6.63 ± 0.41	949.6 ± 41.9	11.0 ± 0.7	295.7 ± 8.5
17	Schaftoside (apigenin 6-*C-β*-d-xyloside-8-*C*-arabinoside)	>5,000	75.59 ± 12.12	>5,000	nd	250.30 ± 4.16
18	Luteolin-6-*C-β*-d-glucopyranoside	102.28 ± 6.20	7.10 ± 0.10	506.6 ± 78.3	7.9 ± 2.4	316.6 ± 15.6
19	Vicenin-3 (apigenin 6-*C-β*-d-glucoside-8-*C*-xyloside)	>5,000	74.76 ± 0.92	>5,000	nd	242.90 ± 11.97
21	Neoschaftoside	>5,000	21.45 ± 0.24	>5,000	nd	430.3 ± 8.0
24	Rutin	43.33 ± 1.75	33.09 ± 0.37	205.0 ± 12.2	10.3 ± 2.8	222.03 ± 13.49
25	Isoquercitrin	197.30 ± 42.07	10.67 ± 0.03	520.2 ± 17.9	15.4 ± 3.7	212.7 ± 10.4
26	Apigenin-6-*C-α*-l-arabinoside-8-*C-β*-d-xyloside	>5,000	80.36 ± 0.77	>5,000	nd	272.7 ± 4.7
27	Quercetin 3-*O*-(6″-*O*-malonyl)-*β*-d-glucoside	24.70 ± 0.96	28.36 ± 0.47	826.7 ± 98.5	12.5 ± 2.9	288.2 ± 17.9
28	Kaempferol-3-*O*-rutinoside	>5,000	35.01 ± 0.22	>5,000	nd	374.4 ± 18.1
31	Astragaline (kaempferol 3-*β*-d-glucopyranoside)	>5,000	34.31 ± 0.10	>5,000	nd	275.7 ± 33.0
32	Isorhamnetin-3-*O*-glucoside	2,366.67 ± 87.61	44.71 ± 1.52	2,757.3 ± 103.8	535.3 ± 150.0	209.2 ± 3.1
Positive	Trolox	158.87 ± 4.47	145.67 ± 1.22	559.8 ± 19.4		
	Celecoxib				89.7 ± 6.4	
	Aminoguanidine hydrochloride					1,604.1 ± 104.5

nd, not detected; values represented as mean ± standard deviation (n = 3); values in the same columns followed by different letters are significantly different at p ≤ 0.05 according to Tukey’s test.

In view of the limitations of different antioxidant methods, the use of at least two or more assays with different mechanisms of oxidation is strongly recommended. Three *in vitro* assays (DPPH, ABTS, and FRAP) were performed to comprehensively analyse the antioxidant capacities of selected compounds (39). These examined compounds demonstrated various influences on these functional activities. All compounds were observed to display significant scavenging capacities against the ABTS radical, with IC_50_ values ranging from 6.63 ± 0.41 μM (compound 16) to 96.39 ± 2.11 μM (compound 14). However, as for the DPPH radical and FRAP assay, some compounds (compounds 10, 14, 17, 19, 21, 26, 28, and 31) showed a low level of inhibition with IC_50_ values that were even higher than 5,000 μM, whereas other compounds (compounds 11, 15, 16, 18, 24, 25, and 27) exhibited extraordinary inhibition, with IC_50_ of DPPH varying from 24.70 ± 0.96 to 226.93 ± 47.26 μM and EC_1_ of FRAP ranging from 205.0 ± 12.2 to 1,100.3 ± 138.4 μM, respectively. The remaining compound 32 had a relatively weak inhibition, with an IC_50_ of DPPH of 2,366.67 ± 87.61 and an EC_1_ of FRAP of 2,757.3 ± 103.8. Based on previous reports, flavonoids appear to exhibit anti-inflammatory properties through the modulation of ROS (40). According to our findings, our data from the anti-COX-2 assay indeed revealed a similar tendency, which was in line with the results observed in the DPPH radical and FRAP assays. Compounds 11, 15, 16, 18, 24, 25, and 27 showed great COX-2 inhibition, with the lowest IC_50_ being 7.9 ± 2.4 μM, as opposed to no inhibitory effects of compounds 10, 14, 17, 19, 21, 26, 28 and 31. Similar to the antioxidant results (DPPH and FRAP) of compound 32, its inhibitory effect on COX-2 was weak with an IC_50_ of 535.3 ± 150.0 μM.

Analysis of the influence on AGE formation illustrated that all compounds were observed to display excellent anti-AGE effects, with IC_50_ ranging from 209.2 ± 3.1 to 374.4 ± 18.1 μM. All the results are very close to that of rutin (IC_50_, 222.03± 13.49 μM), which is not only the main component of DOF-W extracts but also a well-known AGE inhibitor, and the inhibitory effects of all compounds are even better than aminoguanidine hydrochloride (1,604.1 ± 104.5 μM), another frequently used synthetic AGE inhibitor.

## 4 Discussion

In the current study, the results demonstrated that DOF-W exhibited promising antioxidant capacity (DPPH, ABTS, FRAP, intracellular ROS level in NHEK cells), anti-COX-2 effect, anti-glycation potency (inhibition of non-enzymatic glycation reaction and inhibition of CML expression in fibroblasts), and anti-aging effect (SA-*β*-gal staining test and collagen expression in fibroblasts). In addition, chemical and cellular anti-glycation activity as well as the anti-COX-2 effect on DOF-W were reported for the first time.

Oxidative stress and inflammation caused by unstable free radicals, which are highly deleterious to cells and skin, are both major contributors to the aging process (Wang, 2021). Compared with younger skin, elderly skin is more susceptible to environmental stimuli and needs external support such as antioxidants. It is demonstrated that DOF-W had obvious antioxidant and anti-inflammatory effects based on chemical, enzymatic, and cellular methods. Previous studies indicated that DOF-W could increase the antioxidant status and inhibit the inflammatory response in alcohol-impaired mice (Wu et al., 2020), which is consistent with our experimental results. Online UPLC-PDA-MS-ABTS·+ scavenging results indicated that seven compounds (5, 10 or 11, 16, 24, 25, and 27) are most likely to be major contributors to the overall antioxidant potential of DOF-W extracts. However, although all selected compounds displayed an excellent capacity to scavenge ABTS radicals, only compounds 11, 15, 16, 18, 21, 24, 25, and 27 showed great antioxidant potency with respect to DPPH and FRAP assays. Due to the absence of standard 5, its authentic antioxidant capacity cannot be verified in this paper. Combining the results of online and traditional chemical methods, compounds 11, 16, 24, 25, and 27 were predicted to be the main antioxidants of DOF-W extract. Based on previous reports, flavonoids appear to exhibit anti-inflammatory properties *via* the modulation of ROS (40). The anti-cyclooxygenase-2 assay was indeed observed to reveal a similar tendency as the results of the DPPH radical and FRAP assays. Only compounds 11, 15, 16, 18, 21, 24, 25, and 27 showed outstanding COX-2 inhibitory effects. According to our study, the antioxidant and anti-inflammatory effects of DOF-W extract are more likely to be attributed to compounds 11, 15, 16, 18, 24, 25, and 27. Luteolin *C*-glycosylflavones such as compounds 11, 15, and 18 demonstrated great antioxidant and COX-2 inhibitory effects. On the other hand, though apigenin, as one of the most widely distributed flavonoids in the plant kingdom and most frequently studied by researchers, is characterized as a fantastic free-radical scavenger and a remarkable anti-inflammatory agent (41), apigenin *C*-glycosylflavones such as compounds 10, 14, 17, 19, 21, and 26 exhibited very weak biological effects. Among these compounds, compounds 11 and 15 possess the same glycosidic bond as compounds 14 and 19, respectively. Therefore, it looks like that the type of aglycone rather than *C*- or *O*-glycosides of flavonoids, had a great effect on antioxidant and COX-2 inhibitory potency. It has also been reported that both luteolin and apigenin *C*-glycosylflavones had much lower inflammatory effects than those observed with their corresponding aglycones and *O*-glycosides in LPS-induced RAW264.7 (42). Our results may be explained by the speculation that *C*-glycosylation of apigenin leads to a reduction of antioxidant and anti-inflammatory potential. Since most of the flavonoids in plants exist primarily as *O*-glycosides, *C*-glycoside flavonoids received relatively less attention than flavonoid *O*-glycosides, especially in the absence of comprehensive studies on their biological benefits. It is more purposeful to explore the pharmacokinetic properties of flavonoid *C*-glycosides and their bioactivities.

Numerous studies have also shown that external stimuli such as excessive free radicals and spontaneous AGE generation *in vivo*, which are irreversible once formed in the body (11), are associated with skin fibroblast damage, destruction of collagen and elastic fibres, a yellow complexion without splendour, and aging deterioration (12). DOF-W extract displayed excellent capacity to inhibit AGE formation with low IC_50_ values and decreased CML expression obviously in MGO-induced fibroblasts. In addition, all selected compounds were observed to have a potent capacity to inhibit AGE formation. Therefore, anti-glycation ability of DOF-W may be justified by the synergistic action of most polyphenolic compounds present in the extract. So far, several studies have reported that various flavonoids, both *O*- and *C*-glycosides, can cause a marked decrease on AGE production in several *in vitro* and *in vivo* experimental models (43, 44).

Human skin fibroblasts, which primarily exist in the dermis, can form a large amount of collagen, which is a key factor in maintaining skin elasticity. Studies have reported that skin aging may be related to the accumulation of aging fibroblasts within our skin (Wlaschek et al., 2021). Both immunofluorescence assay and extracellular ELISA assay showed that DOF-W extracts can increase the expression of collagen type I, but they have no obvious effect on collagen type III. Moreover, DOF-W can also significantly decrease the proportion of d-Gal-induced senescent cells. In previous research, it had been reported that the *Dendrobium officinale* flower can alleviate brain aging and improve spatial learning abilities in senescent rats (23). Continuous efforts should be made for the detailed identification of representative bioactive constituents in DOF, which may be followed by a systematic clinical study on suitable animal models and humans.

## 5 Conclusion

**Figure 9:**
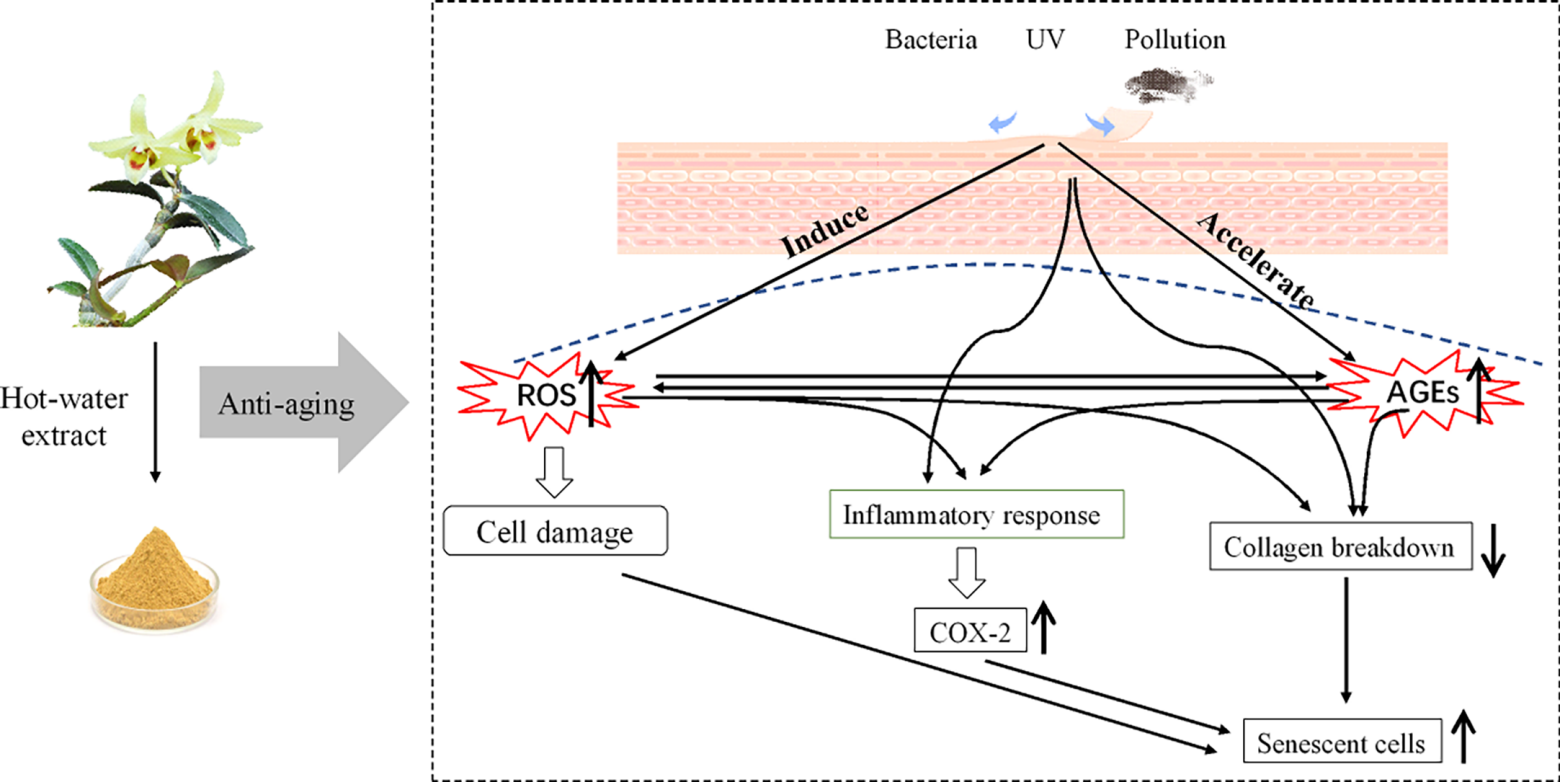
Graphic review of the anti-aging effect of *D. officinale* flower aqueous extract.

Based on the current research regarding *in vitro* studies and phytochemistry analysis, it is suggested that DOF-W is potent with antioxidation, anti-glycation, and anti-aging effects (**Figure 9** **)** and deserves further research and development. Both DOF-W and its specific compounds might be promising agents for skin anti-aging.

## Data availability statement

The datasets presented in this study can be found in online repositories. The names of the repository/repositories and accession number(s) can be found below: MTBLS6933 (MetaboLights).

## Author contributions

Data curation: HZ and RY. Funding aquisition: RY. Project administration: LZ. Supervision: BL and RY. Validation: HZ. Writing original draft: HZ. Writing review and editing: HZ and RY. All authors contributed to the article and approved the submitted version.
