# Supplementary material for: Anti-cyclooxygenase, anti-glycation, and anti-skin aging effect of Dendrobium officinale flowers’ aqueous extract and its phytochemical validation in aging
Source: Front Immunol. 2023 Mar 17;14:1095848. doi: 10.3389/fimmu.2023.1095848 (PMC10064984; doi:10.3389/fimmu.2023.1095848)
Supplement: Supplementary file 1 [file DataSheet_1.docx]

*Determination of Phytochemicals by UPLC-Q/TOF-MS/MS*

Peak **4** with the formula C_13_H_16_O_8_ was identified as salicylic acid 2-*O*-*β*-D-glucoside due to the molecular ions at m/z 299 [M-H]-, accompanied by a characteristic fragment at m/z 137 [M-H-162]- from the loss of a glucose reside as previously reported [[1](#_ENREF_1)]. Compared to peak **5** (1-*O*-caffeoyl-*β*-D-glucoside) that absorbed at 330 nm, peak **6** and peak **7** had similar absorbance spectra with λ_max_ at 314 nm. Based on the MS/MS analysis in negative mode, compound **6** and compound **7** exhibited same molecular ion at m/z 325 and produced fragment ions at m/z 163 (loss of a hexose), 145, 117, and 59, which is characteristic for 1-*O*-(4-Coumaroyl)-*β*-D-Glucose and its isomer. The identification of the molecule was also found to be consistent with published data [[2](#_ENREF_2)].

Flavonoids, especially flavone *C*-glycosides, are the major active constituents of *D. officinale*. Flavone *C*-glycoside, which was different from flavone *O*-glycoside, usually produces neutral loss of characteristic fragments of 90 Da, 120 Da and 150 Da [[2](#_ENREF_2)]. Fifteen flavone *C*-glycosides were identified in this study (compounds **9**, **10**, **11**, **12**, **14**, **15**, **17**, **18**, **19**, **20**, **21**, **22**, **26**, **29,** and **30**). As shown in Table 1, though compounds **10** and **20** exhibited similar molecular ions at m/z 593 [M-H]-, compound **10** produced extra fragment ions at m/z 473 [(M-H)-120]- and 353 [(M-H)-120]-, indicating losses of glucose residues at the 6-position and 8-position [[2](#_ENREF_2)]. Thus compounds **10** was apigenin-6,8-di-*C*-glucoside, also known as vicenin-2, which was further confirmed with authentic standards. Compound **20** was characterised as glucosyl-vitexin, owing to the direct loss of 162 Da and an aglycone radical ion at m/z 431. Fragment ions at m/z 311([M-H-162-120]-) suggested a hexose residue was substituted in compound **20**, which was in accordance with reported data [[3](#_ENREF_3)]. The peaks **14**, **17**, **19,** and **21** exhibited the similar molecular ions at m/z 563 [M-H]−. Compounds **14**, **17**, **19,** and **21** generated the similar fragment ions at m/z 383 and 353, which were characteristic of apigenin-6, 8-di-*C*-glucosides. For the fragment ion m/z 503 [(M-H)-60]−,m/z 473 [(M-H)-90]− and m/z 443 [(M-H)-120]−, suggesting a glucose and pentose residue at the 6-position and 8-position optionally. By comparing to the fragmentation of the reference standards and the published data [[2](#_ENREF_2); [4](#_ENREF_4)], the compound corresponding to peaks **14**, **17**, **19,** and **21** were proposed as apigenin-6-*C*-*β*-D-xyloside-8-*C*-*β*-D-glucoside (also known as vicenin-1), apigenin 6-*C*-*β*-D-glucoside-8-*C*-*α*-L-arabinoside (also known as schaftoside), apigenin-6-*C*-*β*-D-glucoside-8-*C*-*β*-D-xyloside (also known as vicenin-3) and apigenin 6-*C*-*β*-D-glucoside-8-*C*-*β*-L-arabinoside (also known as neoschaftoside), respectively. A typical MS spectrogram fragmentation mechanism for flavonoid disaccharide *C*-glycoside such as schaftoside was shown as fig 6. Compound **22** produced the molecular ion at m/z 533 [M-H-]−, the fragment ion at m/z 443 [(M-H)-90]− and m/z 383 [(M-H)-90-90]− suggested a 6,8-di-*C*-pentose substitution pattern. Based on literature data [[5](#_ENREF_5)]and comparison to reference, compound **22** was confirmed to be apigenin-6-*C*-*β*-D-xyloside-8-*C*-*α*-L-arabinoside. Applying a similar method, compound **26** was tentatively assigned to be apigenin-6-*C*-*α*-L-arabinoside-8-*C*-*β*-D-xyloside by comparing with authentic standard and examining the known flavonoids in different parts of *D. officinale*. Both compounds **29** and **30** exhibited same molecular ion at m/z [M+H]+ 565, and possessed same characteristic fragment ion at m/z 385 [(M+H)-180]+, which implied the loss of a glucose reside. Thus, peaks **29** and **30** were deduced as apigenin-6-*C*-*α*-L-arabinosyl-(1→2)-*O*-*β*-D-glucoside and apigenin-8-*C*-glucosyl-(1→2)-*α*-L-arabinoside, respectively [[5](#_ENREF_5)]. Compounds **9**, **11**, **12**, **15,** and **18** were all preliminarily identified as luteolin *C*-glycosides. MS spectrum of **18** showed [M-H]- ion at m/z 447, and MS/MS fragment ions appeared at m/z 357 and 327, corresponding to [M-H-90]- and [M-H-120]- ions. By comparing with MS of a reference standard and previously reported data, compound **18** was identified as luteolin-6-*C*-*β*-D-glucoside [[6](#_ENREF_6); [7](#_ENREF_7)]. Compared with reference standards, compounds **9**, **11,** and **15** were assigned to be luteolin-6-*C*-*β*-D-glucoside-8-*C*-*β*-D-galactoside, luteolin-6-*C*-*β*-D-xyloside-8-*C*-*β*-D- glucoside and luteolin-6-*C*-*β*-D-glucoside-8-*C*-*β*-D-xyloside. Compound **12** has same [M-H]- ion at m/z 579 and similar MS/MS fragment ions with compounds **11** and **15**. Consequently, compound **12** can just be tentatively deduced as isomer of luteolin-6-*C*-xyloside-8-*C*-glucoside in the absence of a standard.

Flavone *O*-glycoside was also a prominent flavonoid in DOF extract. Compounds **8**, **16**, **23**, **24**, **25**, **27,** and **33** were assigned to be quercetin glycosides by their product ions at m/z 300 and m/z 301 in negative MS2 spectra. The kinds of sugars conjugated with flavonoid aglycone are easily assigned by their fragment ion information in MS^2^ spectrum, which result from the losses of 162 Da for a hexose (a glucose or a galactose), 146 Da for a rhamnose, and 132 Da for a pentose (an apiose, an arabinose, or a xylose) or 324 Da for a sophoroside residue. Compared with the standards, compounds **16**, **23,** and **24** were determined to be quercetin-3-*O*-sophoroside, quercetin-3-*O*-rutinoside (rutin) and quercetin 3-*O*-*β*-D-glucoside (isoquercetrin), respectively. Compound **8** gave a molecular ion [M-H]- (m/z = 771), and fragments of 609, 463, and 301was attributed to the loss of [M-H-162], [M-H-308] and [M-H-162-308]- units, fitting the loss of glucose, rutinose, and both, respectively. MS spectrum of compound **23** showed the [M-H]- ion at m/z 609, which was less 162 Da than that of compound **8**. Compound **23** possessed the similar MS/MS fragments at m/z 463 and 301. As a result, compound **8** and **23** was tentatively assigned to be quercetin 3-*O*-glucosyl-rutinoside and quercetin-7-*O*-rutinoside. Compound **27** produced [M-H-]− at m/z 549 and fragment ions at m/z 505, 463, 301, 300, 271, and 255 which was consistent with previous literature [[8](#_ENREF_8)].Thus, compound **27** was tentatively proposed as quercetin-3-*O*-(6''-malonyl-glucoside). The ion at 505 indicated loss of CO_2_. Ion at 463 was the result of loss of a neutral fragment malondiacyl group with 86 Da. Molecular ion 549 lost the neutral malonyl-glucose fragment of 248 Da to form quercetin ion fragment m/z 301. Fragment ion 255 was produced by the loss of H_2_O and CO from 301 fragment ion. Similar to compound **27**, compound **33** displayed the [M-H]- ion at m/z 506, which was less 44 Da than that of compound **27**. Compound **33** was then tentatively deduced as quercetin 3-*O*-(6''-acetyl-glucoside). Compounds **28** and **31** was identified as kaempferol-3-*O*-rutinoside and kaempferol-3-*O*-glucoside (also known as astragalin). Thus, compound **34** may be malonyl-glucoside of kaempferol, which presented similar fragmentation pathway to that of compound **27**. So, compound **34** was tentatively identified as kaempferol 3-*O*-(6′′-malonyl-glucoside). Compared with the standard, compound **32** deprotonated molecule [M-H]- at m/z 477 and was identified as isorhamnetin-3-*O*-glucoside. Similarly, compound **13** lost C_12_H_20_O_9_ to yield the ion of aglycone at m/z 315. Additionally, the product ion at m/z 357 was generated by the cleavage of glycosyl [[9](#_ENREF_9)]. Compounds **13** was tentatively assigned to be isorhamnetin-3-*O*-neohesperidoside.


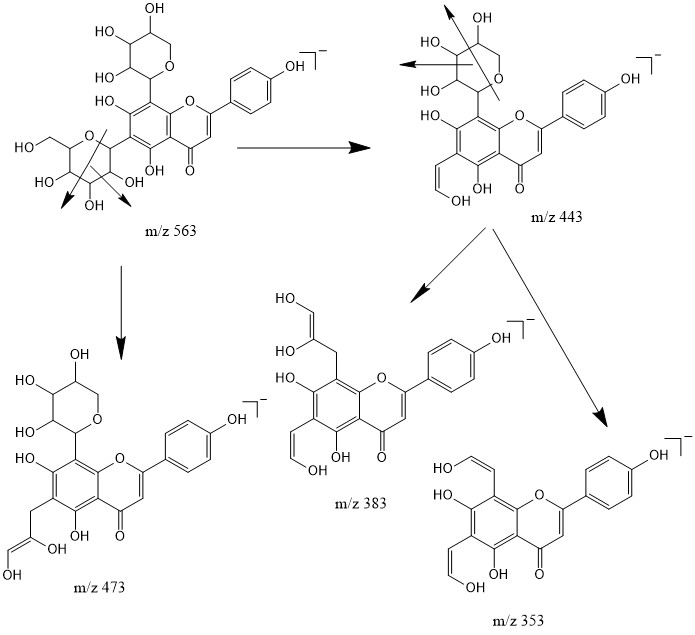


**Supplementary Figure 1**. MS fragmentation mechanism proposed for apigenin 8-C-α-L-arabinoside 6-C-β-D-glucoside (schaftoside).


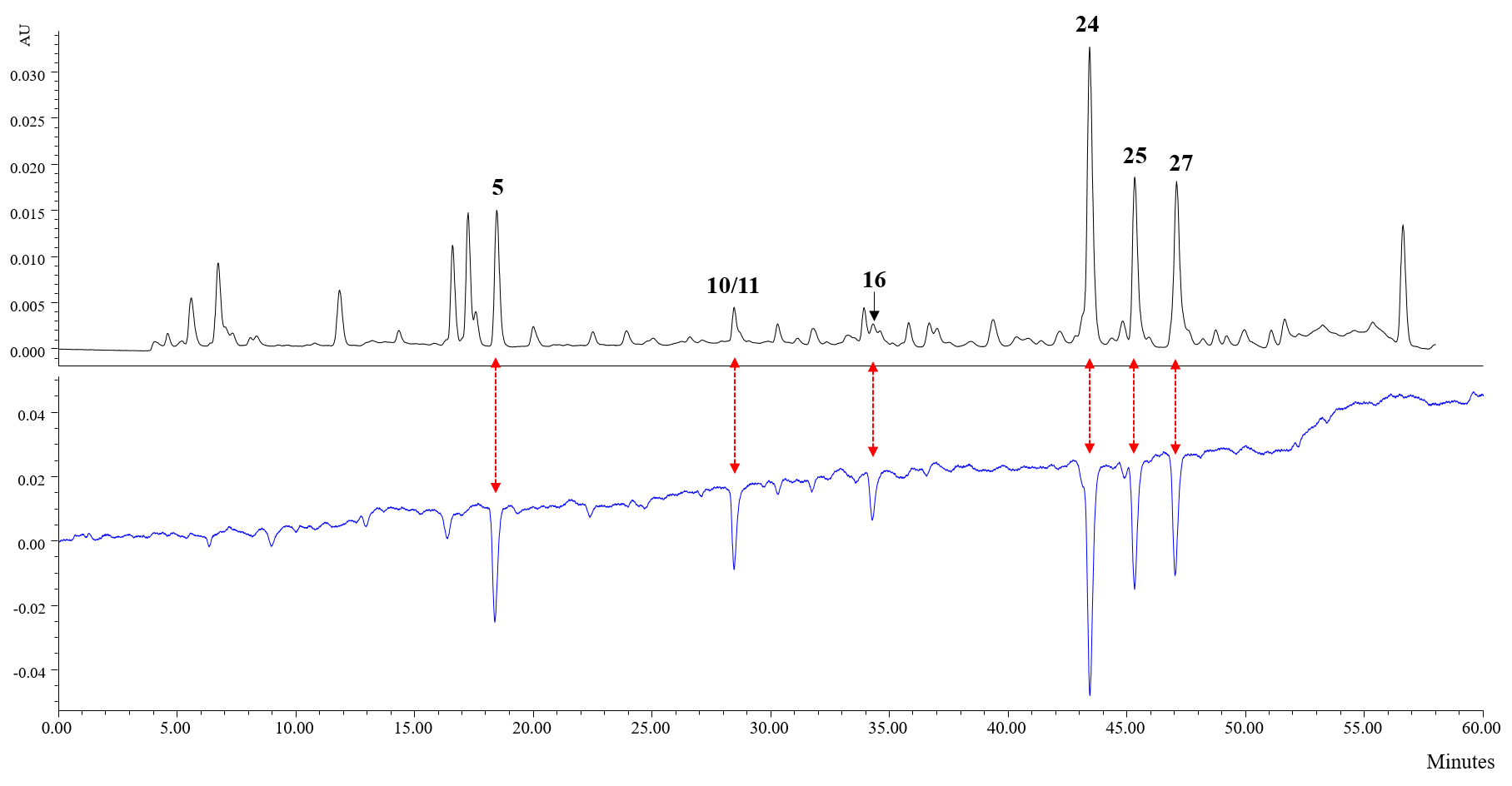


**Supplementary Figure 2**. UPLC-PDA-Qda traces at 254 nm and online antioxidant detection at 734 nm basic component of DOF aqueous extract. Compounds 5, 10, 11, 16, 24, 25, and 27 was 1-O-caffeoyl-β-D-glucoside, vicenin-2, luteolin-6-C-β-D-xyloside-8-C-β-D-glucoside, quercetin-3-O-sophoroside, rutin, isoquercitrin, and quercetin 3-O-(6’’-O-malonyl)-β-D-glucoside, respectively.

**REFERENCES**

[1] R. Shakya, and D.A. Navarre, Rapid screening of ascorbic acid, glycoalkaloids, and phenolics in potato using high-performance liquid chromatography. Journal of agricultural and food chemistry 54 (2006) 5253-60.

[2] X. Zhang, S. Zhang, B. Gao, Z. Qian, J. Liu, S. Wu, and J. Si, Identification and quantitative analysis of phenolic glycosides with antioxidant activity in methanolic extract of Dendrobium catenatum flowers and selection of quality control herb-markers. Food research international (Ottawa, Ont.) 123 (2019) 732-745.

[3] Z. Ren, X. Ji, Z. Jiao, Y. Luo, G.Q. Zhang, S. Tao, Z. Lei, J. Zhang, Y. Wang, Z.J. Liu, and G. Wei, Functional analysis of a novel C-glycosyltransferase in the orchid Dendrobium catenatum. 7 (2020) 111.

[4] Z.Y. Liang, J.Y. Zhang, and Y.C. Huang, Identification of flavonoids in Dendrobium huoshanense and comparison with those in allied species of Dendrobium by TLC, HPLC and HPLC coupled with electrospray ionization multi-stage tandem MS analyses. 42 (2019) 1088-1104.

[5] Z. Ye, J.R. Dai, C.G. Zhang, Y. Lu, L.L. Wu, A.G.W. Gong, and H. Xu, Chemical Differentiation of Dendrobium officinale and Dendrobium devonianum by Using HPLC Fingerprints, HPLC-ESI-MS, and HPTLC Analyses. 2017 (2017) 8647212.

[6] D. Chen, Y. Meng, Y. Zhu, G. Wu, J. Yuan, M. Qin, and G. Xie, Qualitative and Quantitative Analysis of C-glycosyl-flavones of Iris lactea Leaves by Liquid Chromatography/Tandem Mass Spectrometry. Molecules 23 (2018).

[7] G.Y. Xie, Y. Zhu, P. Shu, X.Y. Qin, G. Wu, Q. Wang, and M.J. Qin, Phenolic metabolite profiles and antioxidants assay of three Iridaceae medicinal plants for traditional Chinese medicine "She-gan" by on-line HPLC-DAD coupled with chemiluminescence (CL) and ESI-Q-TOF-MS/MS. Journal of pharmaceutical and biomedical analysis 98 (2014) 40-51.

[8] C. Makita, L. Chimuka, P. Steenkamp, E. Cukrowska, and E. Madala, Comparative analyses of flavonoid content in Moringa oleifera and Moringa ovalifolia with the aid of UHPLC-qTOF-MS fingerprinting. South African Journal of Botany 105 (2016) 116-122.

[9] L.Y. Du, M. Zhao, J.H. Tao, D.W. Qian, S. Jiang, E.X. Shang, J.M. Guo, P. Liu, S.L. Su, and J.A. Duan, The Metabolic Profiling of Isorhamnetin-3-O-Neohesperidoside Produced by Human Intestinal Flora Employing UPLC-Q-TOF/MS. Journal of chromatographic science 55 (2017) 243-250.
